# Quasi-Neutral Theory of Epidemic Outbreaks

**DOI:** 10.1371/journal.pone.0021946

**Published:** 2011-07-08

**Authors:** Oscar A. Pinto, Miguel A. Muñoz

**Affiliations:** 1 Departamento de Física, Instituto de Física Aplicada, Universidad Nacional de San Luis - CONICET, San Luis, Argentina; 2 Departamento de Electromagnetismo y Física de la Materia, Facultad de Ciencias, Instituto de Física Teórica y Computacional Carlos I, Universidad de Granada, Granada, Spain; Massey University, New Zealand

## Abstract

Some epidemics have been empirically observed to exhibit outbreaks of all possible sizes, i.e., to be scale-free or scale-invariant. Different explanations for this finding have been put forward; among them there is a model for “accidental pathogens” which leads to power-law distributed outbreaks without apparent need of parameter fine tuning. This model has been claimed to be related to self-organized criticality, and its critical properties have been conjectured to be related to directed percolation. Instead, we show that this is a (quasi) *neutral model*, analogous to those used in Population Genetics and Ecology, with the same critical behavior as the voter-model, i.e. the theory of accidental pathogens is a (quasi)-neutral theory. This analogy allows us to explain all the system phenomenology, including generic scale invariance and the associated scaling exponents, in a parsimonious and simple way.

## Introduction

Many natural phenomena such as earthquakes, solar flares, avalanches of vortices in type II superconductors, or rainfall, to name but a few, are characterized by outbursts of activity. These are typically distributed as power-laws of their size, without any apparent need for fine tuning – *i.e.* they are generically scale invariant– [1]–[6]. This is in contrast to what occurs in standard criticality, where a control parameter needs to be carefully tuned to observe scale invariance. The concept of *self-organized criticality*, which generated a lot of excitement and many applications in different fields, was proposed to account for generic scale invariance, i.e. to explain the “ability” of some systems to self-tune to the neighborhood of a critical point [1]–[3].

The spreading of some epidemics, such as meningitis in human populations, has been repeatedly reported to exhibit scale-invariant traits, including a wide variability of both durations and sizes of outbreaks. Moreover, the ratio of the variance to the mean of the distribution of meningitis and measles outbreak sizes have been empirically found to be very large and to grow rapidly with population-size [7]. This is the hallmark of anomalously large fluctuations such as those characteristic of the heavy tails of power-law distributions [8]. Actually, power-laws have been proposed to fit the statistics of some epidemics such as measles, pertussis or mumps in some specific locations as the Faroe islands or Reykjavik for which accurate long-term epidemiological data are available [9]–[12]. Remarkably, in some cases, more than four orders of magnitude of scaling have been found [7]. Other features of scale invariance have been reported for measles [7] and other infectious child diseases [13], [14], rabies and bovine tuberculosis [15], or cholera [16].

At a theoretical level, as pointed out by Rhodes and Anderson[9], [10], the lack of a characteristic scale in epidemic outbreaks is reminiscent of earthquakes and their associated (Guttenberg-Richter) power-law distribution. Actually, the presence of scale-invariance in measles, pertussis and others has been justified in [12] by exploiting the analogies between simple models for such epidemics and well-known (self-organized) earthquake models [1]–[3]. In what follows, we focus now on meningitis, for which a related self-organized mechanism has been recently proposed [17]–[22].

The bacteria responsible for meningitis, *Neisseria meningiditis* or meningococcus, is a human commensal: it is typically harmless and it is present in up to one fourth of the human population [23]. Infection is transmitted through close contact with previously infected individuals. It is noteworthy that killing their hosts is a highly undesirable outcome for bacteria; therefore it makes sense that evolution selected for hardly harmful bacteria strains. Nevertheless, the meningococcus can *accidentally* mutate into a potentially dangerous strain, becoming highly damaging or even lethal for the host. This is an example of a more general type of “accidental pathogens” that innocuously cohabit with the host but that eventually –even if rarely– mutate causing symptomatic disease [23].

Aimed at modeling accidental pathogens and to shed some light on the reasons for the emergence of scale invariance in meningococcal epidemics, Stollenwerk, Jansen and coworkers proposed a simple and elegant mathematical model [17]–[22]. The **Stollenwerk-Jansen (SJ) model** is a variant of the basic susceptible-infected-recovered-susceptible (SIRS) model. In the SIRS, individuals can be in any of the following states: “susceptible” (S), “infected” (I), or “recovered” (R) (also called “immune”) [24]–[26]. Perfectly mixed populations are usually considered, i.e. every individual is neighbor of any other (which is a mean-field assumption in the language of Statistical Physics or a “panmictic” one in the Ecology jargon). The additional key ingredient introduced in the SJ model is a second, potentially dangerous, strain of infected individuals labeled 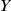. Dangerous strains appear at a very small (mutation) rate at every contagion event. The dynamics of 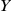 is almost identical to that of 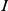 except for the fact that at a certain rate 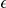 they can cause meningococcal disease of newly infected hosts and eventually kill them, 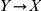 (see below) [17]–[22].

By working out explicitly the analytical solution of this mean-field model as well as performing computer simulations, the authors above came to the counterintuitive conclusion that the smaller the value of 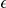 the larger the total amount of individuals killed on average in a given outbreak [17], [18]. This apparent paradox is easily resolved by realizing that the total number of individuals infected with 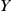 grows upon decreasing 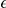. Actually, it has been shown that, whilst for high pathogenicity 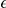 the distribution of the number of observed 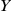-cases, 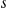, is exponential, in the limit 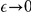 it becomes power-law distributed, obeying
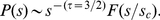
(1)where 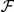 is some scaling function and 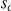 a maximum characteristic scale controlled by 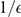 [17], [18].

Observe that the exponent 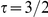 matches that of a critical branching process, for which the average population of infected individuals does not either grow or decay in time but stays constant [27]. Also, 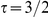 coincides with the exponent for the distribution of first return times of an unbiased random-walk [28], which describes generically the scaling of avalanches in mean-field models.

However, the value 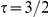 predicted by the SJ model in its mean-field version, does not necessarily correspond to the best fit to empirical data [7], [9], [10]. To scrutinize the possible origin of such discrepancies, one would like to go beyond the mean-field/panmictic hypothesis by considering structured populations in which each individual has a finite local neighborhood. In what follows we shall study the SJ model on populations distributed on regular (Euclidean) lattices in dimensions 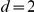 and 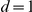. The study of more complex networks (as small-world networks or networks with communities), aimed at describing more realistically the net of social contacts, is left for a future work.

As already pointed out [17], [18], the SJ model can be straightforwardly made spatially explicit. Even if analytical or numerical calculations have not been performed, it was conjectured in [17]–[20] that in 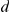-dimensions the SJ model should be in the *directed percolation* class, i.e. the broad and robust universality class characterizing phase transitions between an active and an absorbing state [29]–[34]. In the present case, the absorbing state would be the 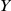-free state obtained for sufficiently small 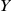-infection rates, while the active or endemic phase would correspond to a non-vanishing density of 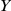
's. In particular, if the critical behavior was indeed that of directed percolation, then 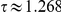 for two-dimensional populations (and 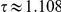 for epidemics propagating in one dimension) (see [35] and [36]).

A priori, the prediction of directed percolation scaling is somehow suspicious if the model is indeed self-organized; it has been shown that in general models of self-organized criticality, as sandpiles, ricepiles, earthquake models, etc. are *not* in the directed percolation class [37]–[41]. Furthermore, models of self-organized criticality lacking of any conservation law (as is the case of the SJ model) have been shown not to be strictly critical, i.e. they are just approximately close to critical points [42]; instead the exact solution by Stollenwerk and Jansen proves that their model is exactly critical [17], [18]. These considerations cast some doubts on the conjecture of the SJ model being a model of self-organized criticality [1], [2], [37]–[41]. Therefore, one is left with the following open questions: what is the SJ model true critical behavior?, what is the key ingredient why accidental pathogens – as described by the SJ model– originate scale invariant outbreaks?, does such an ingredient appear in other epidemics?

The purpose of the present paper is to answer these questions by analyzing the SJ model in spatially extended systems.

## Methods

The spatially explicit SJ model is defined as follows. Each site of a 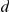-dimensional lattice is in one of the following states 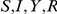, or 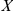. The dynamics proceeds at any spatial location 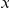 and any time 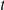 according to the following one-site processes:

- Spontaneous recovery of the benign strain: 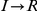, at rate 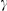.
- Spontaneous recovery of the dangerous strain: 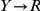, at rate 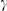.
- Loss of immunity: 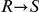 at rate 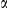.
- Replacement or recovery of diseased: 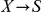, at rate 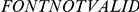,

and two-site processes:

- Infection with the benign strain: 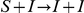, at rate 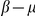.
- Infection with the dangerous strain: 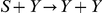, at rate 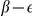.
- Mutation: 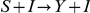, at rate 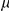.
- Disease: 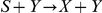, at rate 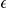.

Some comments on these reaction rules are in order. Accordingly to [21] it is assumed that being infected with one strain protects against co-infection with a second one. This assumption, which is supported by some empirical observations [21], prevents transitions as 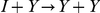 from appearing. 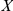 (diseased/dead) individuals are immediately replaced by new susceptible ones, so that the total population size is kept fixed; i.e. 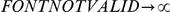. A “back mutation” (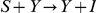) reaction could be introduced, but for most purposes its rate is so small that it can be neglected. The dynamics is easily implemented in computer simulations by using the Gillespie's algorithm [43] or a variation of it in a rather standard way. In the well-mixed case writing the densities of the different species as 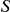, 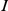, 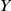, 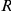, and 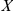, one readily obtains the following mean-field or rate equations:
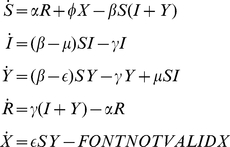
(2)satisfying the constraint 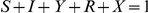. Stollenwerk and Jansen [17], [18] worked out the exact solution of this set of equations, concluding that it is critical in the limit 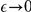, and obtaining the explicit form of Eq.(1).

Instead, in spatially explicit models this mean-field approach breaks down: (i) the densities need to be replaced by spatio-temporal fields, as 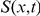, 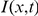, 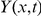, etc; (ii) new (Laplacian) terms describing the map of nearest-neighbor contacts appear; (iii) fluctuations become relevant and noise terms need to be added to account for demographic fluctuations. A full set of such stochastic Langevin equations can be derived from the microscopic dynamics by using standard procedures [28], [44], [45] and numerically investigated [46] (results not shown here).

## Results

We now report on extensive numerical simulations for the SJ model. We have chosen the following parameter values: 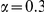, 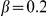 and 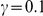, and have tried other different sets to confirm the robustness of the results. We consider the mutation rate 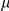 to be sufficiently small, such that strains generated by consecutive mutations do not overlap; i.e. outbreaks finish well before a new mutation appears. For this reason we take as initial condition for any outbreak a state with a single mutant of the potentially dangerous strain 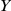 and effectively fix 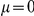 during outbreaks.

### Two dimensions

We consider square lattices of linear size 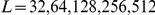, and 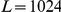, take a random initial condition with only 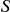 and 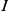 individuals, and run the dynamics with periodic boundary conditions, keeping 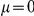, until a steady state is reached. For instance, for 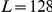, the steady state is characterized by 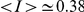, 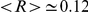 and 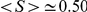 for the set of parameters above.

Once the system sets into its steady state, we place a 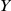 individual at the geometrical center of the lattice and study its spreading [31]–[34]. To avoid finite size effects, spreading experiments are stopped once the 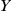-strain touches the system boundary, and the described procedure is iterated. Depending on system size we ran up to 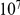 independent realizations. As customarily done, we monitor: (i) the epidemics size distribution, analogous to Eq.(1), for both 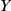 and 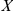; (ii) the average total number of 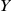 as a function of time 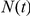; (iii) the surviving probability 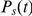 that the 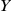 strain is still present in the system at time 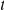, and (iv) the average square radius from the origin of 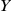-infected individuals, 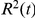. At criticality, these quantities are expected to scale algebraically as 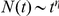, 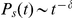, and 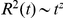, while they should show exponential cut-offs in the sub-critical (or absorbing) phase.

**Figure 1:**
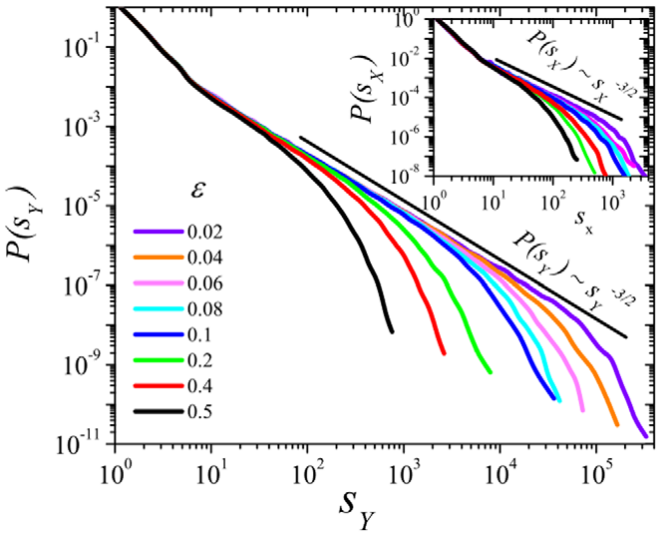
Main: Avalanche size distribution, 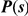 for various values of 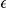 in a two dimensional lattice of size size 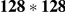, and associated distribution for avalanche sizes of 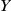-infected individuals, 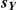 (main plot), and 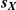 of 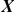-infected ones (inset).

Fig. 1 shows the epidemic outbreak size distribution for different values of 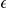 and system size 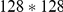 (size of 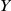-infected outbreaks, 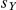, in the main plot, and size of 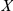-infected outbreaks, 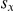, in the inset). The probability distribution of avalanches sizes 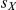 for 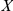-infected sites is observed to inherit the statistics of 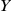-infected ones. Both distributions can be well fitted by Eq.(1) where 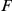 is a cut-off (exponential) function and 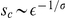 determines the maximum size. The best fit gives 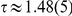 (fully compatible with 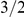 as shown in Fig. 1) and 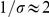 (not shown) both for 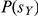 and 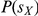. From these plots we conclude that the system becomes critical in the limit of vanishing 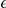, as occurs in mean-field. Observe that for small pathogeneicities, as 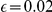, the system, even if sub-critical, exhibits scaling along more than three orders of magnitude.

**Figure 2:**
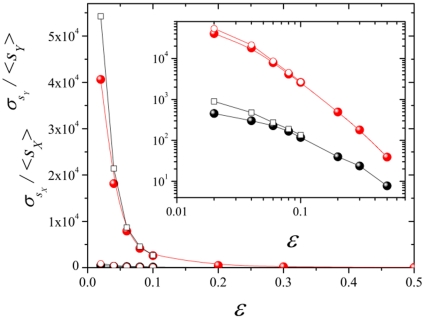
Ratio of the variance (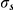) to the mean size (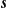) for 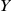 (red) and for 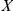 (black), for and different values of and system sizes 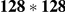 (full symbols) and 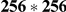 (empty symbols). The ratio diverges in the double limit 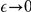, 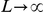. Inset: as the main plot, but in double logarithmic scale: as system-size increases the curves converge to straight lines, i.e. power-laws, but finite size effects are significant.

Both the mean and the variance of the above distributions are observed to diverge as power-laws in the limit 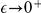. Actually, as shown in Fig. 2 the ratio of the variance 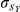 (resp. 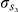) to the mean 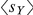 (resp. 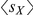) diverges when 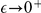 as 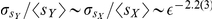 in the infinitely large system-size limit (otherwise, for finite 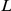 a size-induced cut-off appear as illustrated in Fig. 2 by comparing simulations for two different sizes).

Analogously, fixing 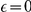 we measure the mean and variance as a function of system size; the ratio of the variance to the mean diverges very fast as 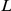 grows, 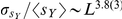 (results not shown). Observe that, trivially, in this case 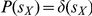, i.e. no death is produced.

**Figure 3:**
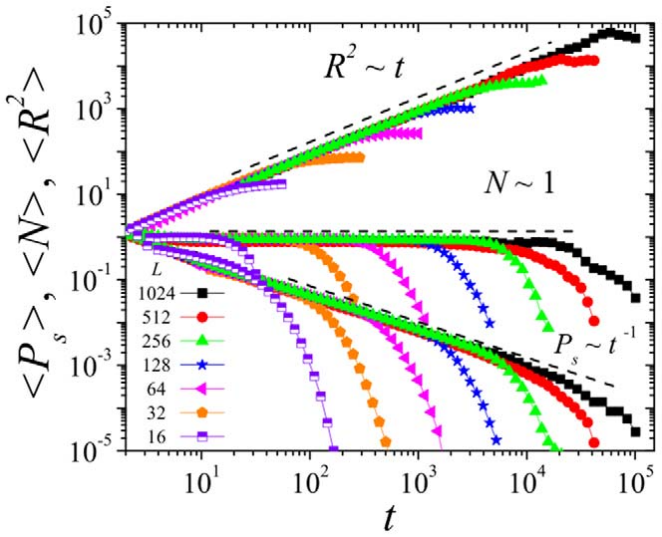
Log log plot of 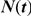, 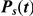, and 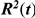, for spreading experiments in different two-dimensional systems (linear sizes 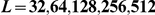, and 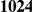) as a function of time 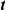. Dashed lines are a guide to the eye.

**Figure 4:**
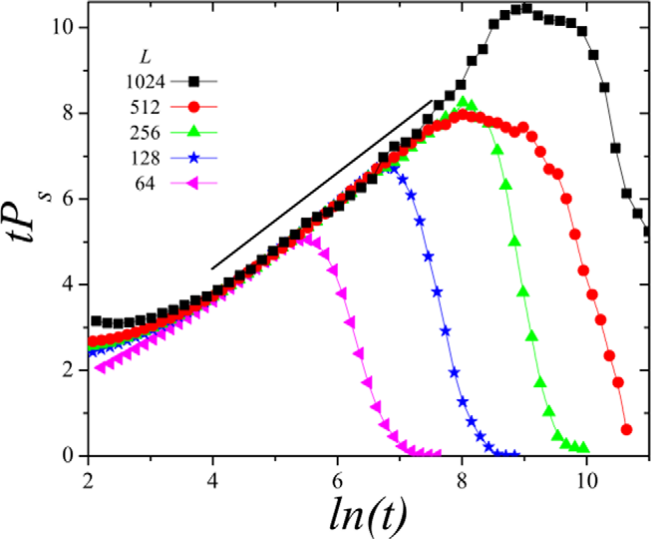
Plot of 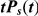 as a function of 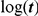 for different system sizes (from 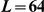 to 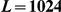), illustrating the presence of linear logarithmic corrections for the surviving probability in the two-dimensional SJ model.

Fig. 3 shows results obtained for spreading quantities for various sizes and 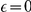, i.e. at criticality. The best fits we obtain for the asymptotic behavior of these three magnitudes are
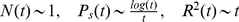
(3)An effective power-law, with exponent slightly smaller than unity can be fit to our numerical results for 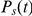 (see Fig. 3); however, such an effective value of the exponent can be seen to grow upon extending the maximum time, making the case for a logarithmic correction as described by Eq.(3) and illustrated in Fig. 4. Observe also that the reported values, 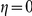, 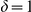, 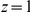 satisfy the hyper-scaling relation 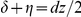 using 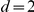 [35].

Contrarily to a priori expectations and to a previous conjecture, these asymptotic laws have nothing to do with directed percolation values (which predicts pure power law behavior for the three spreading quantities [31]–[34] with 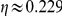, 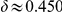, 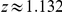, 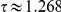; see [35] for a set of spreading and avalanche exponent numerical values in different universality classes).

Instead, our results for spreading are in excellent agreement with the expectations for the two-dimensional voter-model [47]–[49] universality class (also called “compact directed percolation class” [31]–[33], [50]) [34], [35], [51] (see below). Also, our results for the size distribution are in excellent agreement with the two-dimensional voter class expectations, 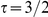 and 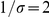. In particular, using these theoretical values, the variance to mean ratio should scale as 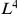 in good agreement with the numerical finding above.

### One dimension

To further confirm the conclusion above, we have also performed studies of one-dimensional lattices, for which the expected behavior in the voter-model class is:
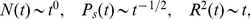
(4)while the avalanche size exponent is 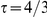 [35].

**Figure 5:**
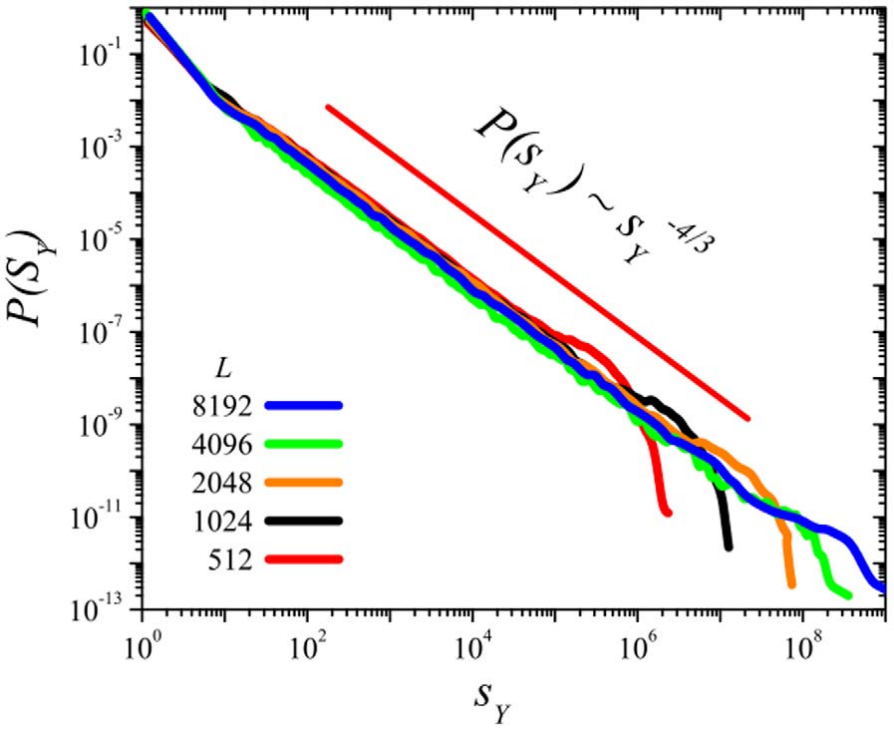
Avalanche size distribution, 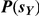 for 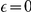 and various one-dimensional lattices, of size 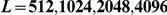, and 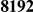, respectively. The straight line corresponds to the theoretical prediction for the one-dimensional voter-model class.

**Figure 6:**
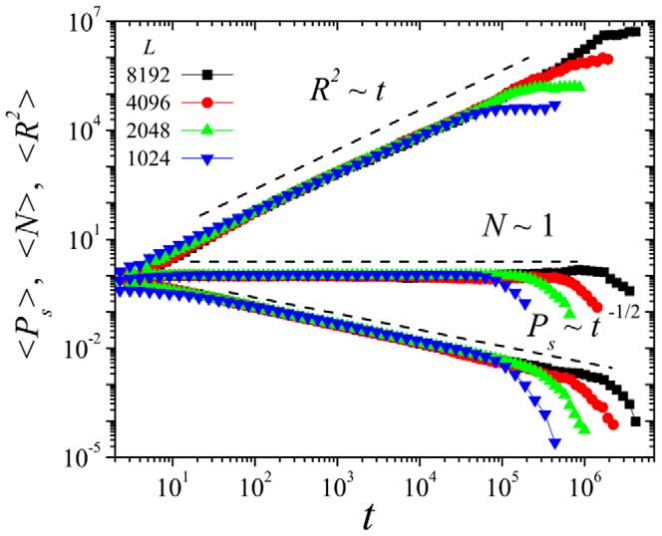
Evolution of 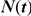, 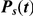, and 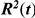 as a function of time 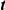 in log-log scale for spreading experiments performed on one-dimensional lattices of linear sizes 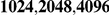, and 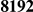. Parameter values are 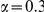, 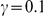, and 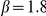. Results are averaged over 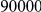 independent realizations.

Fig. 5 shows the avalanche size distribution for various one dimensional sizes (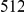, 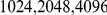, and 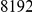) and 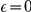. The measured 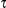 exponent, 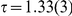 is in excellent agreement with the voter-model class expectation. Fig. 6 shows the result of spreading experiments (for 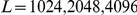, and 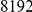). The best fits to the slopes, 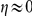, 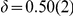, and 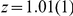 are in excellent agreement with the theoretical prediction (see dashed lines in Fig. 6). These results confirm that the SJ model is in the voter-class also in one spatial dimension (and exclude one-dimensional directed percolation values 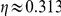, 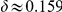, 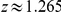, 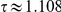).

## Discussion

Neutral theories date back to the sixties when Kimura introduced them in the context of population genetics [52]. Kimura assumed, as a null or neutral model, that each allele of a given gene (in haploid populations) is equally likely to enter the next generation, i.e. allele-type does not affect the prospects for survival or reproduction. Inspired in this, Hubbell proposed an analogous neutral theory of (forest) bio-diversity, in which the prospects of death and reproduction do not depend on the tree species [53]. Both theories lead to correct predictions and also to interpretation controversies (see [54] for a recent review).

In its simplest spatially explicit version the neutral theory is as follows. Consider, for argument's sake, the neutral theory of bio-diversity with only two tree species. It can be formulated by considering the spatially explicit Moran process [55] or *voter model* [34], [47]–[49]. This is defined by two symmetrical species (up/down, right/left, black/white, etc.) fully occupying a 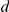-dimensional lattice (or, more in general, an arbitrary network). At some rate a randomly chosen individual is removed and replaced by any of its nearest neighbors with homogeneous probability. This leads to a local tendency to create clusters of any of the two symmetrical species. Clusters of each of the two species will occupy different positions until eventually one of the two species will take over the whole (finite) space, leading to fluctuation induced mono-dominance in any finite system-size.

Such a type of coarsening process has been studied in depth; the asymptotic scaling of the spreading quantities, 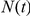, 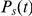, and 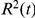 is analytically predicted to be given by Eq.(3). In particular, the logarithmic corrections for the surviving probability correspond to the fact 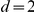 is the upper critical dimension of the voter universality class and are closely related to the marginality of the return time of two-dimensional random walkers [36], [47]–[49]. The key ingredients of this universality class turn out to be the symmetry between the two existing absorbing (mono-dominated) states [51], [56], [57] and the absence of surface tension [51].

It is important to underline that, given the symmetry (neutrality) between the two species in the voter model, a given population of any of the species can either grow or decline with the same probability; i.e. there is no deterministic bias. Using the field theoretical jargon, the “mass” or “gap” term vanishes; all this is tantamount to the model being critical [58].

Indeed, for the voter model in mean-field, calling 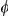 the density in one of the two states (and 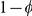 the complementary density for the second species), then
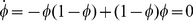
(5)where the first term represents the loss of an individual in the first state by contact with a neighboring in the second one, and the second represents the reverse process. Therefore, the average density of 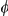 is constant all along the system evolution. Actually, in the voter-model there is no control parameter to be tuned; the model lies, by definition at a critical point: criticality is imposed by the neutral symmetry. Instead, introducing a bias or preference towards one of the species,
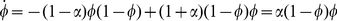
(6)where the constant 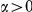 quantifies the degree of asymmetry, a non-vanishing linear or “mass” term is generated, bringing about an overall tendency for 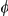 to grow or to decrease depending on the sign of 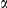, i.e. deviating the system from criticality.

Introducing spatial dependence and stochasticity, Eq.(5) transforms into the Langevin equation for the voter class [51]

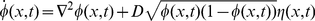
(7)where 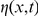 is a Gaussian white noise (observe that the equation is symmetrical under the change 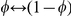 and that there are two absorbing states at 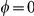 and 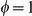, respectively).

Despite of the coincidence in the asymptotic scaling, let us underline that the SJ model is *not* a voter model. In the SJ model there are 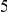 different species and not just 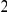. In the case 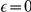, however, 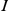 and 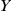 are perfectly symmetrical; but as processes as 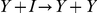 or 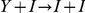 do not exist, replacement of one species by the other occurs only if mediated by 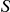 particles. It is, therefore, only at sufficiently coarse grained scales that the dynamics behaves as the voter-model; microscopically the two dynamics differ significantly.

Even if the SJ is not a voter model, the underlying reason for it to exhibit scale-invariance is that, in the limit 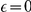 and 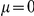 the model is “neutral” (i.e. strains 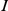 and 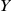 are perfectly symmetrical) and, as a direct consequence, it is critical. More explicitly, calling 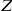 the total density of infected sites which includes both 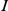 and 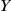 then, at a mean field level, keeping 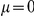 and 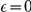


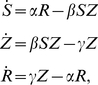
(8)and the steady state is 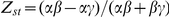, 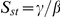, and 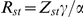. Let us suppose, for argument sake, that 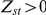; i.e. that there is a non-vanishing stationary density of infected sites (otherwise epidemics would just extinguish in finite time) and that at time 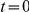 is 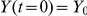. The evolution of 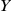 is controlled by
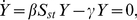
(9)implying 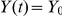, i.e. there is no bias for 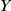 to grow or decay, or in other words, under these conditions the model is critical in what respects the 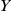 variable. For example, in spreading experiments we fix 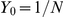 (and 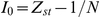), implying that the total number of 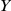-sites is 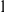 on average, in agreement with the numerical findings in Fig. 3 and Fig. 6. Switching on a non-vanishing 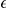 is equivalent in the voter model to introduce a bias, inducing deviations from criticality, i.e. exponential cut-offs for the size distribution and spreading quantities, as indeed observed in our numerical simulations for 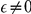 (see, for instance, Fig. 3 and Fig. 6).

It is noteworthy that in the perfectly symmetrical (neutral) case, 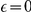, 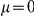, the model is somehow dull: two benign strains compete in a critical way, but there is no observable consequence of this for the population under study. The interesting behavior of the SJ model comes from slight deviations from criticality, this is, 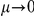 and 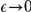 with 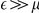; it is in this double limit that outbreaks leave behind an observable scale-invariant distribution of sick/dead individuals.

In summary, the Stollenwerk-Janssen model provides us with the most parsimonious explanation for the appearance of scale-invariant epidemic outbreaks caused by accidental pathogens such as the meningococcus. Our main finding is that the system is critical in the limit of vanishing pathogenicity in all dimensions, and the reason for this is that the SJ is a *neutral model*, which turns out to be critical for the very same reasons as other neutral theories in Population Genetics and Ecology are critical. As a consequence of this, the critical behavior of the model is *not* described by directed percolation as previously conjectured, but instead by the voter-model universality class, representative of neutral theories. Understanding the theory of accidental pathogens as a neutral theory gives us new insight into the origin of generic scale invariance in epidemics in particular and in propagation phenomena in general.

Similar ideas might apply to related problems as the spreading of computer viruses in the Internet or in the web of e-mail contacts: the largest overall damage is expected to occur for any type of spreading agents if their probability to cause damage is as small as possible. Being close to neutrality warrants success on the long term.

## References

[1] Bak P, Tang C, Wiesenfeld K (1987). Self-Organized Criticality: An Explanation of 1/*f* Noise.. Phys Rev Lett.

[2] Bak P (1996). How Nature works: The science of self-organized criticality.. Copernicus.

[3] Jensen HJ (1998). Self-Organized Criticality.

[4] Grinstein G (1995).

[5] Grinstein G (1991). Generic Scale Invariance in Classical Nonequilibrium Systems.. J Appl Phys.

[6] Dickman R, Muñoz MA, Vespignani A, Zapperi S (2000). Paths to Self-Organized Criticality.. Braz J Phys.

[7] Keeling M, Grenfell B (1999). Stochastic dynamics and a power law for measles variability.. Phil Trans R Soc Lond B.

[8] Newman MEJ (2005). Power laws, Pareto distributions and Zipf's law.. Contemp Phys.

[9] Rhodes CJ, Anderson RM (1996). Power laws governing epidemics in isolated populations.. Nature.

[10] Rhodes CJ, Anderson RM (1996). A scaling analysis of measles epidemics in a small population.. Philos Trans R Soc Lond B Biol Sci.

[11] Rhodes CJ, Butler AR, Anderson RM (1998). Epidemiology of communicable diseases in small population.. J Mol Med.

[12] Rhodes CJ, Jensen HJ, Anderson RM (1997). it On the critical behaviour of simple epidemics.. Proc R Soc London Ser B.

[13] Trottier H, Philippe P (2005). Scaling properties of childhood infectious diseases epidemics before and after mass vaccination in Canada.. J Theor Biol.

[14] Philippe P (2000). Epidemiology and self-organized critical systems: An analysis in waiting times and diseases heterogeneity.. Nonlinear Dyn Psychol Life Sci.

[15] Harnos A, Reiczigel J, Rubel F, Solymosi N (2006). Scaling properties of epidemiological time series.. Appl Ecol and Environmental research.

[16] Azaele S, Maritan A, Bertuzzo E, Rodriguez-Iturbe I, Rinaldo A (2010). Stochastic dynamics of cholera epidemics.. Phys Rev E.

[17] Stollenwerk N, Jansen VAA (2003). Meningitis, pathogenicity near criticality: the epidemiology of meningococcal disease as a model for accidental pathogens.. J Theor Biol.

[18] Stollenwerk N, Jansen VAA (2003). Evolution towards criticality in an epidemiological model for meningococcal disease.. Phys Lett A.

[19] Stollenwerk N (2005). Self-organized criticality in human epidemiology in Modeling Cooperative Behavior in the Social Sciences.. AIP Conf Proc.

[20] Stollenwerk N (2005). Criticality in Epidemics: The Mathematics of Sandpiles Explains Uncertainty in Epidemic Outbreaks.. Recent Advances in Applied Probability.

[21] Stollenwerk N, Maiden MCJ, Jansen VAA (2004). Diversity in pathogenicity can cause outbreaks of meningococcal disease.. Proc Natl Acad Sci U S A.

[22] Guinea F, Stollenwerk N, Jansen VAA (2005). Statistics of infections with diversity in the pathogenicity.. Biophysical Chemistry.

[23] Maiden MCJ (2000). High-throughput sequencing in the population analysis of bacterial pathogens of humans.. Int J Med Microbiol.

[24] Anderson RM, May RM (1992). Infectious Diseases of Humans: Dynamics and Control..

[25] Hethcote HW (2000). The Mathematics of Infectious Diseases.. SIAM REVIEW.

[26] Murray JD (1993). Mathematical Biology..

[27] Harris TE (1989). The theory of branching Processes.

[28] Gardiner CW (1985). Handbook of Stochastic Methods..

[29] Grassberger P (1982). On phase transitions in Schoegl's second model Z.. Phys B.

[30] Janssen HK (1981). On the nonequilibrium phase transition in reaction-diffusion systems with an absorbing stationary state.. Z Phys B.

[31] Hinrichsen H (2000). Nonequilibrium Critical Phenomena and Phase Transitions into Absorbing States.. Adv Phys.

[32] Henkel M, Hinrichsen H, Lübeck S (2008). Non-equilibrium Phase transitions..

[33] Ódor G (2004). Universality Classes in Nonequilibrium Lattice Systems.. Rev Mod Phys.

[34] Marro J, Dickman (2005). R Non-equilibrium phase transitions in lattice models.

[35] Muñoz MA, Dickman R, Vespignani A, Zapperi S (1999). Avalanche and spreading exponents in systems with absorbing states.. Phys Rev E.

[36] Zillio T, Banavar JR, Green JL, Harte J, Maritan A (2008). Incipient criticality in ecological communities.. Proc Natl Acad Sci U S A.

[37] Vespignani A, Dickman R, Muñoz MA, Zapperi S (1998). Driving, Conservation and Absorbing States in Sandpiles.. Phys Rev Lett.

[38] Vespignani A, Dickman R, Muñoz MA, Zapperi S (2000). Absorbing Phase Transitions in Fixed-Energy Sandpiles.. Phys Rev E.

[39] Bonachela JA, Ramasco JJ, Chaté H, Dornic I, Muñoz MA (2006). Sticky grains do not change the universality of isotropic sandpiles.. Phys Rev E.

[40] Bonachela JA, Muñoz MA (2007). How to discriminate easily between Directed-percolation and Manna scaling.. Physica A.

[41] Bonachela JA, Muñoz MA (2008). Confirming and extending the hypothesis of sandpile universality.. Phys Rev E.

[42] Bonachela JA, Muñoz MA (2009). Self-organization without conservation: true or just apparent scale-invariance?. J Stat Mech.

[43] Gillespie D (1977). Exact stochastic simulation of coupled chemical reactions.. Jour of Phys Chem.

[44] Doi M (1976). Second quantization representation for classical many-particle system.. J Phys A.

[45] Peliti L (1985). Path-integral approach to birth-death processes on a lattice.. J Physique.

[46] Dornic I, Chaté H, Muñoz MA (2005). Integration of Langevin Equations with Multiplicative Noise and Viability of Field Theories for Absorbing Phase Transitions.. Phys Rev Lett.

[47] Ligget TM (1985). Interacting particle systems..

[48] Bramson M, Cox JT, Durrett R (1996). Spatial models for species area curves.. Ann Probab.

[49] Bramson M, Cox JT, Durrett R (1998). A spatial model for the abundance of species.. Ann Probab.

[50] Domany E, Kinzel W (1984). Equivalence of Cellular Automata to Ising Models and Directed Percolation.. Phys Rev Lett.

[51] Dornic I, Chaté H, Chave J, Hinrichsen H (2001). Critical Coarsening without Surface Tension: The. Universality Class of the Voter Model.. Phys Rev Lett.

[52] Kimura M (1983). The Neutral Theory of Molecular Evolution..

[53] Hubbell SP (2001). The Unified Neutral Theory of Biodiversity and Biogeography..

[54] Leigh EG (2007). Neutral theory: a historical perspective.. Journal of Evolutionary Biology.

[55] Moran PAP (1962). The Statistical Processes of Evolutionary Theory..

[56] Al Hammal O, Chaté H, Dornic I, Muñoz MA (2005). Langevin description of critical phenomena with two symmetric absorbing states.. Phys Rev Lett.

[57] Dickman R, Tretyakov AYu (1995). Hyperscaling in the Domany-Kinzel cellular automaton.. Phys Rev E.

[58] Amit DJ, Martn Mayor V (2005). Field Theory, the Renormalization Group and Critical Phenomena..

